# The Effects of an Arbuscular Mycorrhizal Fungus and Rhizobium Symbioses on Soybean Aphid Mostly Fail to Propagate to the Third Trophic Level

**DOI:** 10.3390/microorganisms10061158

**Published:** 2022-06-04

**Authors:** Élisée Emmanuel Dabré, Jacques Brodeur, Mohamed Hijri, Colin Favret

**Affiliations:** 1 Institut de Recherche en Biologie Végétale, Département de Sciences Biologiques, Université de Montréal, 4101 rue Sherbrooke Est, Montréal, QC H1X 2B2, Canada; jacques.brodeur@umontreal.ca (J.B.); mohamed.hijri@umontreal.ca (M.H.); colinfavret@aphidnet.org (C.F.); 2 African Genome Center, Mohammed VI Polytechnic University (UM6P), Lot 660, Hay Moulay Rachid, Ben Guerir 43150, Morocco

**Keywords:** *Aphis glycines*, *Bradyrhizobium japonicum*, co-inoculation, multitrophic interaction, *Rhizophagus irregularis*, soybean aphid, natural enemy, symbiosis

## Abstract

The cascading effects of microbe–plant symbioses on the second trophic level, such as phytophagous insects, have been most studied. However, few studies have examined the higher third trophic level, i.e., their natural enemies. We investigated the effects of the symbiotic associations between an arbuscular mycorrhizal (AM) fungus, *Rhizophagus irregularis* (Glomerales: Glomeraceae), a nitrogen-fixing bacterium, *Bradyrhizobium japonicum* (Rhizobiales: Bradyrhizobiaceae), and soybean, *Glycine max* (L.) Merr. (Fabaceae) on two natural enemies of the soybean aphid, *Aphis glycines* Matsumura (Hemiptera: Aphididae), the ladybird beetle *Coleomegilla maculata* (De Geer) (Coleoptera: Coccinellidae), and the parasitoid *Aphelinus certus* Yasnosh (Hymenoptera: Aphelinidae). We measured the growth and survival in the predator and parasitoid reared on aphids feeding on soybean inoculated seedlings. The rhizobium symbiosis alone was affected with a decreased rate of parasitoid emergence, presumably due to decreased host quality. However, number of mummies, sex-ratio, development time, and parasitoid size were all unaffected by inoculation. AM fungus alone or co-inoculated with the rhizobium was unaffected with any of the parameters of the parasitoid. For the predator, none of the measured parameters was affected with any inoculant. Here, it appears that whatever benefits the microbe–plant symbioses confer on the second trophic level are little transferred up to the third.

## 1. Introduction

Interactions between plants, herbivores, and their natural enemies, such as predators and parasitoids, are important factors of trophic cascades and play a major role in community diversity and functioning [1,2]. Plant size, vigor, and primary and secondary chemistry are important mediators of the interactions between herbivores and their natural enemies [3,4]. The mechanisms underlying these tritrophic interactions may be affected by microbial root mutualists acting on variable plant traits [5]: qualitative and quantitative effects induced by these belowground organisms can affect aboveground multitrophic interactions [6,7,8,9]. Arbuscular mycorrhizal (AM) fungi and Rhizobia are two of the most important and widely studied belowground microbes associated with plant roots [5,10,11]. Arbuscular mycorrhizal fungi form symbiotic associations with the roots of most terrestrial plants [12]. During symbiosis, plants provide carbon for the AM fungus, and in return the fungus transfers nutrients, particularly phosphates in phosphorus-poor conditions [13,14]. Rhizobia are bacteria that are associated with most legumes with which they form root nodules and enhance nitrogen uptake via N_2_ fixation [15]. Therefore, legumes such as soybean can form beneficial tripartite symbiotic associations with AM fungi and rhizobia simultaneously [16,17,18].

These rhizosphere interactions can also affect the abundance of herbivorous insects associated with the aerial parts of plants [4,19]. Studies have shown that AM fungi can also influence insects at higher trophic levels, namely, the predators [6,20,21] and parasitoids of the herbivores [22,23,24,25]. For example, the inoculated sweet pepper with AM fungus significantly increased the density of ladybird predators of the peach potato aphid, *Myzus persicae* (Sulzer) (Hemiptera: Aphididae), in the field [26]. However, no clear effects were seen in the greenhouse; even the plant–AM fungus symbiosis reduced colonization by the aphid [26]. Another study showed a significant decrease in the population growth of the aphid *Rhopalosiphum padi* Linnaeus (Hemiptera: Aphididae) and an increased rate of parasitism in aphids by the parasitoid wasp *Aphidius rhopalosiphi* DeStefani-Perez (Hymenoptera: Braconidae), even when no clear effects of AM fungus on N and P concentration were observed in the plant’s foliage [24]. These authors suggested there might be complex, but yet unknown, species-specific cascading effects of AM fungus in the food chain. Furthermore, rhizobia associated with plants has been shown to affect parasitoids [27,28] and predators [29], but it seems that the results in all these studies were context-dependent.

In addition, prey quality determines the fitness of predators and parasitoids [4]. There is a need to better understand these interactions, especially with regard to possible biological control agents of herbivorous insects [30,31].

Belonging to the second trophic level, phloem-feeding aphids can be influenced by AM fungi [32,33,34,35,36] and Rhizobia [37,38] colonizing the roots of their host plants. However, the direction of the effects has varied between experiments, the microbial symbionts sometimes improving sometimes harming herbivore performance [39]. The nutritional quality of a host plant, and especially the low level of nitrogen available in its phloem, significantly limits aphid growth, including that of the soybean aphid [40]. In a previous study, Dabré et al., in revision [41] showed that the inoculation of AM fungus and Rhizobia in soybean increased the population growth of soybean aphid, *Aphis glycines* Matsumura (Hemiptera: Aphididae), likely following an increase in the concentrations of N and P in the plant. Here, we extend the work to study how AM fungus and rhizobia might indirectly affect the natural enemies (third trophic level) of soybean aphid.

We studied the effects of a tripartite association between an AM fungus *Rhizophagus irregularis* (Glomerales: Glomeraceae), a rhizobium *Bradyrhizobium japonicum* (Rhizobiales: Bradyrhizobiaceae), and soybean *Glycine max* (L.) Merr. cv. ETNA on two natural enemies of the soybean aphid, the predatory ladybeetle *Coleomegilla maculata* (De Geer) (Coleoptera: Coccinellidae) and the parasitoid *Aphelinus certus* Yasnosh (Hymenoptera: Aphelinidae). We hypothesized that the inoculation of either microbial symbiont alone or their co-inoculation would increase the rate of parasitism on aphids and their fitness, presumably due to an increased nutritional quality in the host. Secondly, for the same reasons, symbionts were expected to increase the performance (weight and development time) of the predator. To test these hypotheses, we performed a growth chamber experiment by using aphids fed on plants inoculated with four inoculants treatments (control without inoculant, AM fungus alone, Rhizobium alone, and mycorrhizal and rhizobial inoculation). Then, we compared different fitness parameters measured on parasitoids (mummification rate, emergence rate, sex ratio, development time) and predators (development time, adult and fourth instar weight) relative to microbial inoculant.

## 2. Materials and Methods

### 2.1. Study Insects

The soybean aphid, *Aphis glycines*, is an invasive pest of soybean that was first detected in the United States in 2000 and quickly became the dominant and significant insect pest in soybean in North America [42,43]. It has a high reproductive capacity and in high numbers can damage the plant sufficiently enough to cause significant yield loss [40,44]. In Quebec, *A. glycines* is only an occasional pest of soybean because its populations do not usually reach threshold levels when the plant is in its most susceptible life stages: vegetative and flowering [45]. Aphid populations are also usually kept under control by natural enemies [46].

In this study, the soybean aphid was provided by the entomological lab of Jacques Brodeur (Dabré et al., in revision) [41]. Individuals were maintained on soybean, *Glycine max*. cv. ETNA in walk-in growth chamber under the following conditions: 60–70% RH, L16:D8 photoperiod, at a constant temperature of 22 °C. Older plants with aphids were replaced weekly by two-week-old plants.

The parasitoid wasp *Aphelinus certus* is a natural enemy of soybean aphid from Asia, introduced accidently in Ontario (Canada) in 2007 [47]. It has since become common throughout the soybean-growing regions of North America [48,49,50]. We established a colony of *A. certus* from mummies collected on soybean plants at Saint-Hyacinthe, Québec, Canada (45.605° N, 72.916° W) on 13 September 2019. All insect life stages were maintained at 25 °C, 50–60% RH, and an L16:D8 photoperiod. Adult males and females were kept in boxes with soybean plants infested by soybean aphids. After aphid mummification and emergence of adult parasitoids, we changed the plants with other plants infested by aphids. Droplets of 10% glucose (honey) were placed on parafilm and hung inside the box as a food source for the adults.

The pink-spotted lady beetle, *Coleomegilla maculata* (Coleoptera: Coccinellidae), is a relatively polyphagous coccinellid species that is found throughout the Americas [51]. It feeds on a variety of pests including chrysomelid and lepidopteran larvae and eggs, spider mites, and aphids [52,53,54]. In this study, we established a colony of lady beetles collected in a maize field at Saint-Simon, Québec, Canada (45.681° N, 72.858° W) on 20 September 2019. Colony was held in the same conditions as those of the aphid (60–70% RH, L16:D8 photoperiod, at 22 °C). Adults were kept in plastic boxes (24.43 cm length × 16.81 cm width × 8.55 cm height) on cut paper and were fed immature and adult soybean aphids. We brought 10% glucose water and pure water, each provided on a small piece of absorbent cotton in a plastic tube and changed it out every three days. We also provided bee pollen to supply as an additional protein source. Every three days, each food source was renewed. Clusters of eggs, usually laid on the cut paper or on the inner surfaces of the boxes, were collected twice weekly and transferred to 5.5 cm diameter petri dishes until hatching. Larvae were then fed with aphids in Petri dishes to adult stage and transferred in the boxes. After one generation, field-collected adults were removed.

Aphid, parasitoid, and predator voucher specimens were deposited in the Université de Montréal Ouellet-Robert Entomological Collection with catalog numbers QMOR61.147-QMOR61.152, QMOR57.627-57.628, and QMOR57.629-57.633, respectively.

### 2.2. Plant and Microbial Materials

Soybean seeds (cultivar ETNA) and commercial inoculants were supplied by Premier Tech (Rivière-du-Loup, QC, Canada). The mycorrhizal inoculant contains 500 spores per gram of *R. irregularis* isolate DAOM197198. This isolate has been the most prevalent commercially-available Arbuscular Mycorrhizal (AM) fungi for the last two decades [55]. The Rhizobial inoculant was a liquid solution containing *B. japonicum* PTB 162 at a concentration of 7 × 109 colony-forming units per milliliter of inoculum.

### 2.3. Experimental Design

The experiment was conducted under controlled conditions in a walk-in growth chamber. The experiment was set up in a randomized block design with four treatments of microbial inoculant, namely, control without inoculation (M−R−), mycorrhizal inoculant (M+R−), rhizobium inoculant (M−R+), and double inoculant with mycorrhizal and rhizobial (M+R+) (Appendix A; Table A1). Nine and eight replicates per each inoculant treatment, for the parasitoid and the ladybird beetle experiment, respectively, were performed. Four inoculant treatments were added to sterile pots. AM fungus inoculation consisted of 2 g of paper-wrapped commercial inoculum [6] roughly 5 cm below the soil surface [18], 1 cm below the germinated seeds. Inoculation with rhizobia was performed with 40 µL of inoculum placed directly on the germinated seed.

Soybean seeds were germinated on agar medium for three days before sowing. Sowing was carried out in 45–50 mL germinating cells with potting substrate PROMIX-PGX (Premier Tech, Rivière-du-Loup, QC, Canada) containing vermiculite. Two weeks later, the plants were transferred into 3 L pots (6.5′′ × 7′′) on potting soil BERGER BM6, a horticultural coarse sphagnum peat mixture (80–90%) and perlite (Terris, Laval, QC, Canada) (see Dabré et al., in revision) [41]. For the duration of the experiment, soybean plants were kept in a growth chamber at 20 °C (light) and 18 °C (dark), L16:D8 photoperiod, and at 55–65% relative humidity. Constant light was provided by 16 neon tubes of 160 W each. They were fertilized with 50 mL of nutrient Long Ashton solution [56] one time per week and watered twice a week with the same quantity of water.

Each plant enclosed in a box made of a transparent cylindrical rigid plastic 55–60 cm in height and 25 cm diameter and closed at the top with a fine mesh net (30 cm in diameter). Six weeks after transplanting, the plants had attained the third trifoliate stage, and we tested a subset of roots to confirm the establishment of the microbial symbioses in inoculated plants (Dabré et al., in revision) [41]. Four apterous adult aphids were placed on the top of each plant of each treatment to give birth for 48 h. These adults were then removed, and the number of first-instar, same-generation aphid nymphs was standardised at 5.

Two weeks later, 40 s to fourth-instar aphids [57] were randomly collected from each plant of the four inoculants treatments following the same protocol described above and introduced onto a fresh-cut soybean leaf in Petri dishes 10 cm in diameter (9 replicates per each treatment). A mated female parasitoid wasp, 1–2 days old, was introduced into each dish to oviposit on the aphids and removed 24 h later [58]. Petri dishes with parasitized aphids on leaves were kept at 26 °C in the day and 24 °C in the night until mummification [49]. Each mummy was removed and placed in a gelatin capsule and held until emergence of parasitoid in the same conditions. Two visual inspections were made each day, in the morning and evening.

The predator experiment was conducted two weeks after the introduction of aphids. The experiment was carried out in 32 (8 replicates per each treatment) Petri dishes of 10 cm diameter with moistened filter paper. In each Petri dish, a first instar larva was introduced and fed, ad libitum, a mix of aphid life stages, until adult pupation. Two visual inspections were made each day throughout the experiment.

### 2.4. Insects’ Parameters Measured

For parasitoids, the following fitness parameters were recorded: total number of mummified aphids; time between mummification and adult emergence; number of emerged female and male adults; length of the left hind tibia of each emerged parasitoid as a proxy of total insect size [59].

For predators, we recorded the inter-molt duration of each instar and the pupa. At the last instar, we measured the larval weight daily until pupation. Finally, we recorded the emergence time of adults, i.e., the duration of the pupal stage.

### 2.5. Statistical Analysis

Statistical analyses were performed in R [60]. All the data were tested for normality of distribution and homogeneity of variance by Shapiro–Wilk test and Levene’s test, respectively. For parasitoids, to examine the effects of AM fungi and rhizobia inoculation, the same number of aphids from each of the four inoculant treatments replicated nine times are followed to evaluate variables as the number of mummies, the parasitoid’s survival, and the hind tibia length of female and male parasitoids. We performed linear mixed effects model (LMM) with the function lmer in package lme4 [61] and fitted the model with ANOVA. The parasitoid variables, sex ratio and development time, did not meet the assumption of normality and equality of variance using the Kruskal–Wallis test [26], followed by post hoc Dunn’s test [62] for multiple comparisons between inoculant treatments when significant differences were observed.

To assess the influence of the inoculation on ladybird beetles, different parameters such as larval stages, pupation duration, the fourth larval stage, and adult weight were measured on the first larval stage fed with aphids from each of four inoculated plants replicated eight times, as evaluation variables. We also performed the same model as above (LMM) followed by ANOVA. For all the statistics where we performed LMM, inoculation treatments were used as fixed factors (independent variables), and blocks as random factors, and the different parameters measured on parasitoids and ladybird beetles as dependant variables.

## 3. Results

### 3.1. Effects of Soybean Aphid Parasitoid, Aphelinus Certus of AM Fungi, and Rhizobia Inoculation on Soybean

Parasitoid survival was different between inoculants. The rhizobium (M−R+) treatment had the lowest proportion of emerged adult parasitoid, with all three other treatments yielding equivalent or higher survival rates (Table 1). However, any significant difference in aphid parasitism by *A. certus* between the inoculant treatments was observed concerning the number of mummies and the adult wasp size, as measured by hind tibia length (Table 1). In addition, no difference was observed between inoculant treatments in either the adult sex ratio (females on males) or in the wasp development time of females and males (Table 2).

### 3.2. Effects of AM Fungi and Rhizobia Inoculation on Ladybird Beetle, Coleomegilla Maculata

There was no measurable effect of any inoculant on measured fitness parameters of the ladybeetle (Table 3) in terms of duration of the larval stages, pupal stage, and total pre-imaginal life, the weight of the fourth instar, or the weight of the adult emergence.

## 4. Discussion

The only significant result was for the adult parasitoid survival in the rhizobium-only (M−R+) inoculated treatment, where the survivorship of the pupal stage of *A. certus* developing on soybean aphid increased by 42.6% to 54.2% relative to the control, mycorrhiza, and double inoculant (mycorrhiza and rhizobium) (Table 1); no effect on other tested parameters were observed. The negative effect of rhizobium inoculation on adult parasitoid emergence found in our experiment is similar to what was found by Pineda et al. (2013) [28], who documented a decreased abundance of the parasitoid *Diaeretiella rapae* (McIntosh) (Hymenoptera: Braconidae) on the aphid *M. persicae* feeding on *Arabidopsis thaliana* (L.) (Brassicaceae) associated with the bacterium *Pseudomonas fluorescens*. Similar to our results, neither these authors nor Pangesti et al. (2015) found any effect of the bacteria on the development time or weight of the parasitoid [28,29].

Furthermore, they [28] showed that the blend of volatile compounds from rhizobacteria-treated aphid-infested plants is modified and less attractive to the aphid parasitoid than that of a control. In contrast, positive effects of rhizobia-induced volatile on other parasitoid taxa have been documented [4]. For example, Pangesti et al. (2015) [29] documented an increased attraction of the parasitoid *Microplitis mediator* (Haliday) (Hymenoptera: Braconidae) for the caterpillar *Mamestra brassicae* (L.) (Lepidoptera: Noctuidae) on rhizobium-associated plants (*A. thaliana*). After analysis of the volatile compounds, they found, in response to caterpillar feeding, a suppression by the microbial symbiosis of the emission of compounds such as (E)-α-bergamotene (a terpene), methyl salicylate, and lilial (aromatics) [29].

Our study also did not find any effect of AM fungus inoculation on the parasitic wasp (Table 1). In the same line, Gange et al. (2003) [22] presented the first study focusing on AM fungi–herbivore–parasitoid interaction and they found no effects with the parasitoid *Diglyphus isaea* (Walker) (Hymenoptera: Eulophidae) attacking the leaf-miner *Chromatomyia syngenesiae* Hardy (Diptera: Agromyzidae) on plants associated (*Leucanthemum vulgare* Lam.) with AM fungus *Funneliformis caledonium* (syn. *Glomus caledonium* (T.H. Nicolson & Gerd.) Trappe & Gerd. or *Rhizophagus fasciculatus* (syn. *Glomus fasciculatum* (Thaxt.) Gerd. & Trappe)). Likewise, another study showed similar results with no effects of the AM fungus *Claroideoglomus etunicatum* (syn. *Glomus etunicatum* W. N. Becker & Gerdemann) on the parasitism of the silver leaf whitefly (*Bemisia argentifolii* Bellows & Perring) (Hemiptera: Aleyrodidae) by the parasitoid *Eretmocerus eremicus* Rose & Zolnerowich) (Hymenoptera: Aphelinidae) [25]. However, these authors found positive and negative effects of AM fungus on the parasitism rate depending on AM fungi species, with concomitant changes in leaf chemistry.

As mentioned above, as examples, Pineda et al. (2013) [28] showed a negative effect of rhizobia on parasitoid, while Gange et al. (2003) [22] found that AM fungi inoculation helped parasitoid. Then, in our study, the absence of effect with the double inoculation (M+R+) could be explained by the presence of AM fungus: perhaps the negative effects of the rhizobium were mitigated by the presence of AM fungus in the double inoculation.

AM fungi and rhizobia inoculation did not influence any measured parameter of the soybean aphid predator *C. maculata* (Table 3). Previous studies have shown a positive response of predators feeding on prey on AM fungi-induced plants, specifically with an increase of the predator’s population growth rate [20] or, similarly, the predator’s final population abundance [6,21]. Meanwhile, effects induced by rhizobia can be transferred up to the third trophic level: Katayama et al. (2011) documented an increase in the abundance of an aphid predator, the ladybird beetle *Coccinella septempunctata* Linnaeus (Coleoptera: Coccinellidae), on soybean plants inoculated with rhizobia [30].

A critical factor of aboveground tritrophic interactions is prey/host quality affecting the fitness of predators and parasitoids [4]. Another mechanism that affects tritrophic interactions is when beneficial organisms, such as AM fungi and rhizobia, alter the nutrient and toxin concentrations of the tissues of herbivores [63]. In a trophic cascade system, it has been shown that the upper levels have higher concentrations of N and P than do lower, such as plant vs. herbivores and herbivores vs. natural enemies (predators and parasitoids) [4,64]. Thus, an increase in these nutrients in the plant can indirectly enhance predator and parasitoid fitness through that of herbivores [65]. In an AM fungi–plant interaction study, the higher nutritive quality of the two-spotted spider mite *Tetranychus urticae* Koch (Trombidiformes: Tetranychidae) improved the reproduction on its predator, *Phytoseiulus persimilis* Athias-Henriot (Mesostigmata: Phytoseiidae) [20].

Dabré et al. *in revision* [42], studying the same study system, did not document any effect of plant chemical composition (as affected by rhizosphere microbe inoculation) on soybean aphid size, although they did record an increased population growth rate. Similarly, Hempel et al. (2009) [24] showed that the inoculation of AM fungus on timothy grass (*Phleum pratense* L.), albeit decreasing *Rhopalosiphum padi* (L.) (Hemiptera: Aphididae) population growth, increased the rate of parasitism in aphids by the parasitic wasp *Aphidius rhopalosiphi* (De Stefani Perez) (Hymenoptera: Braconidae), but with no clear effects of AM fungus inoculation on the concentration of nitrogen and phosphorus in the aphid’s plant host. They suggested that other cascading effects affect the second (herbivores) and third (parasitoids) trophic levels more than does plant nutritional status alone [24]. In addition, root mutualists may alter the quality of prey or host by impacting plant secondary chemicals sequestered by herbivores to protect them against natural enemies [66]. Another possible explanation concerns insect-associated microbes that can enhance resource acquisition or provide protection against biotic or abiotic factors on their host [67]. Some facultative endosymbiotic bacteria can protect aphids from parasitoids. For example, the secondary symbiont *Hamiltonella defensa* Moran et al. (Gammaproteobacteria: Enterobacteriaceae) can protect the pea aphid *Acyrthosiphon pisum* (Harris) (Hemiptera: Aphididae) from braconid parasitoid wasps [68]. This defensive effect is variable, however: a study showed that two *Aphelinus* species (*A. atriplicis* Kurdjumov and *A. glycinis* Hopper and Woolley) reacted differently when parasitizing aphids infected with *H. defensa* [69]. Not having assessed the nutritional and secondary chemicals, volatile compounds, and the associated-bacterial endosymbionts of soybean aphid, we cannot at present make any conclusions regarding the possible indirect role of microbial mutualists.

In conclusion, this study showed that the mutualistic interaction of soybean plants with AM fungi and Rhizobia mostly failed to influence the third trophic level insects such as parasitoids and predators. However, there was a negative response of parasitoids with a decrease of parasitoid survival in the presence of rhizobium inoculant, presumably due to the intrinsic quality of the herbivore prey. It has been shown that by affecting plant nutritional status, root microbes belowground can indirectly affect predators and parasitoids above ground. Another important factor that can influence the third trophic level insects concerns the induction of the defense mechanisms of plant with the emission of volatile organic compounds (VOCs). In this case, and to better understand the mechanisms mediating microbe–plant–insect relationships, we suggest further study to explore the impact of root beneficial microorganisms on host insect body through changes in nutrients, secondary chemistry, and impact of facultative endosymbionts associated.

## Figures and Tables

**Table 1:** Average of parasitoid wasp parameters measured as variables (mean ± SE, F, df = 3, *p*) of 9 replicates per inoculant treatment (N = 9). Control: M−R−; Mycorrhizae+Rhizobium: M+R+; Mycorrhizae: M+R−; Rhizobium: M−R+. Linear mixed effect model (LMM) follows by ANOVA. Letters followed by mean ± SE represent Tukey’s honest significant difference (HSD) groupings.

Variables	Inoculant Treatments				*F*-Values	*p*
	M−R−	M+R−	M−R+	M+R+		
Number of mummies	10.89 ± 3.46	7.89 ± 2.25	4.33 ± 2.00	4.33 ± 1.36	1.7470	0.1772
Proportion of emerged parasitoids (%)	66.7 ± 11.54 b	78.3 ± 7.77 b	24.1 ± 8.79 a	70.4 ± 10.97 b	6.3826	0.0025
Tibia length-females (µm)	283 ± 9.01	281 ± 4.97	286 ± 9.07	269 ± 9.05	0.8247	0.2909
Tibia length-males (µm)	262 ± 11.50	261 ± 12.87	255 ± 14.52	253 ± 9.08	0.3990	0.9404

**Table 2:** Sex ratio and development time of parasitoid wasp tested as variables by applying Kruskal–Wallis test (*χ*^2^, df = 3, *p*) to differentiate inoculant treatments grouping (N = 9). Control: M−R−; Mycorrhizae+Rhizobium: M+R+; Mycorrhizae: M+R−; Rhizobium: M−R+. *p* > 0.05 (not significant).

Variables	*χ* ^2^	*p*
Sex ratio (F/M)	3.9429	0.2677
Development time of females (days)	5.6273	0.1370
Development time of males (days)	1.1173	0.7729

**Table 3:** The average number of ladybeetles four larval stages and pupation duration, the weight of the fourth larval and adult (mean ± SE, F, *p* values, N = 8) of 8 replicates per treatment. Control: M−R−; Mycorrhizae+Rhizobium: M+R+; Mycorrhizae: M+R−; Rhizobium: M−R+. Linear mixed effect model (LMM) follow by ANOVA was applied, and no measurable difference was observed between the inoculant treatments following the means.

Variables	Inoculant Treatments				*F*	*p*
	M−R−	M+R−	M−R+	M+R+		
L1 duration (days)	2.71 ± 0.16	2.74 ± 0.17	2.74 ± 0.17	2.87 ± 0.13	0.6406	0.4339
L2 duration (days)	2.64 ± 0.15	2.69 ± 0.13	2.78 ± 0.16	2.70 ± 0.16	0.2387	0.6340
L3 duration (days)	3.00 ± 0.00	2.89 ± 0.12	3.17 ± 0.16	3.23 ± 0.17	0.5515	0.4720
L4 duration (days)	5.31 ± 0.17	5.45 ± 0.18	4.83 ± 0.26	5.26 ± 0.47	0.4830	0.5131
Pupation (days)	6.29 ± 0.18	6.00 ± 0.00	6.29 ± 0.18	5.86 ± 0.14	0.3000	0.6004
L4 weight (g)	13.3 ± 0.25	13.6 ± 0.70	14.4 ± 0.57	14.1 ± 0.78	0.7340	0.4266
Adult weight (g)	11.0 ± 0.01	11.9 ± 0.24	12.2 ± 0.26	12.3 ± 0.45	1.8576	0.2285

## Data Availability

Not applicable.

## Appendix A

**Table A1:** Inoculant treatments used in the experiment.

Treatments			
M−R−	M−R+	M+R−	M+R+
Control, neither mycorrhizal nor rhizobial inoculant	Rhizobial inoculant alone	Mycorrhizal inoculant alone	Mycorrhizal and Rhizobial inoculant together

M: Mycorrhiza; R: Rhizobium; −: absence; +: presence.
